# Time Trends in the Incidence of Long-Term Mortality in T2DM Patients Who Have Undergone a Lower Extremity Amputation. Results of a Descriptive and Retrospective Cohort Study

**DOI:** 10.3390/jcm8101597

**Published:** 2019-10-02

**Authors:** Ana López-de-Andrés, Rodrigo Jiménez-García, Maria D. Esteban-Vasallo, Valentin Hernández-Barrera, Javier Aragon-Sánchez, Isabel Jiménez-Trujillo, Javier de Miguel-Diez, Maria A. Palomar-Gallego, Martin Romero-Maroto, Napoleón Perez-Farinos

**Affiliations:** 1 Preventive Medicine and Public Health Teaching and Research Unit, Health Sciences Faculty, Rey Juan Carlos University, 28922 Madrid, Spain; ana.lopez@urjc.es (A.L.-d.-A.); valentin.hernandez@urjc.es (V.H.-B.); isabel.jimenez@urjc.es (I.J.-T.); 2 Department of Public Health & Maternal and Child Health, Faculty of Medicine, Universidad Complutense de Madrid, 28040 Madrid, Spain; 3 Madrid Regional Health Authority, Public Health Directorate, 28040 Madrid, Spain; maria.estebanv@salud.madrid.org; 4 Department of Surgery, Diabetic Foot Unit, La Paloma Hospital, 35005 Las Palmas de Gran Canaria, Spain; javiaragon@telefonica.net; 5 Respiratory Department, Hospital General Universitario Gregorio Marañón, Facultad de Medicina, Universidad Complutense de Madrid, Instituto de Investigación Sanitaria Gregorio Marañón (IiSGM), 28009 Madrid, Spain; javier.miguel@salud.madrid.org; 6 Basic Science Department, Health Sciences Faculty, Rey Juan Carlos University, 28922 Madrid, Spain; mariaangustias.palomar@urjc.es; 7 Medical Department, Health Sciences Faculty, Rey Juan Carlos University, 28922 Madrid, Spain; mromeromaroto.urjc@gmail.com; 8 Department of Public Health and Psychiatry, Faculty of Medicine, Universidad de Malaga, 29071 Malaga, Spain; napoleon.perez@uma.es

**Keywords:** amputations, type 2 diabetes mellitus, mortality, cohort study

## Abstract

(1) Background: The aims of this study were to examine the incidence of lower extremity amputations (LEAs) among patients with type 2 diabetes mellitus (T2DM) and to compare the mortality risk of diabetic individuals who underwent LEA with age and sex-matched diabetic individuals without LEA. (2) Methods: We performed a descriptive observational study to assess the trend in the incidence of LEA and a retrospective cohort study to evaluate whether undergoing LEA is a risk factor for long-term mortality among T2DM patients. Data were obtained from the Hospital Discharge Database for the Autonomous Community of Madrid, Spain (2006–2015). (3) Results: The incidence rates of major below-knee and above-knee amputations decreased significantly from 24.9 to 17.1 and from 63.9 to 48.2 per 100000 T2DM individuals from 2006 to 2015, respectively. However, the incidence of minor LEAs increased over time. Mortality was significantly higher among T2DM patients who underwent LEA compared with those who did not undergo this procedure (HR 1.75; 95% CI 1.65–1.87). Male sex, older age, and comorbidity were independently associated with higher mortality after LEA. (4) Conclusions: Undergoing a LEA is a significant risk factor for long term mortality among T2DM patients, and those who underwent a major above-knee LEAs have the highest risk.

## 1. Introduction

Lower extremity amputation (LEA) is one of the most serious surgical procedures performed in patients with diabetes [1]. Patients with diabetes are more likely to require LEA than people without diabetes [2]. For example, in a national Spanish study between 2001 and 2012, 65.8% of all nontraumatic LEA procedures occurred in patients with diabetes [3].

Amputations in people with diabetes are a result of the combination of several conditions that include as key contributory factors, peripheral vascular disease (PVD), neuropathy (distal sensorimotor peripheral and autonomic), and infection. Neuropathy and PVD often co-exist and may lead to neuro-ischemic ulceration [4,5,6].

PVD independently increases the risk of major non-traumatic LEAs and failure to diagnose and adequately treat underlying PVD is a major cause of amputations in people with diabetes [6]. The prevalence of PVD among people with diabetes has risen steadily throughout the past three decades, and PVD is estimated to be present in as many as 50%–60% of patients with diabetic foot ulcers (DFUs) [5]. Studies have shown that the identification of PVD in patients with DFUs and aggressive, timely revascularization reduces amputation rates [4,5].

Several other conditions are known to be associated with an increased risk of foot ulceration. All stages of nephropathy increase the risk of ulceration, with the highest risk found in the dialysis population. Dialysis treatment is an independent risk factor for foot ulceration [4,5]. Any deformity occurring in a foot with other risk factors increases ulcer risk [4,5]. Clawing of the toes is common, leading to increased metatarsal head pressures that, in neuropathic patients, may result in breakdown due to repetitive moderate stress to an insensate area. Other conditions include Charcot foot (CF) and hallux valgus [4,5]. CF is a fracture dislocation process that affects the bones, joints, and ligaments of the foot and ankle in people with peripheral sensory neuropathy [7]. When it is not detected early, it may result in a secondary ulceration, infection, and amputation. A recent study shows, however, that CF alone may not pose a risk for amputation, but that CF along with ulceration increases the risk of amputation by 12 times [7].

For people with diabetes, diabetic foot infections (DFIs) are the most common diabetes-related reason for hospitalizations and for lower extremity amputations [5]. It is estimated that approximately 20% of moderate or severe diabetic foot infections lead to some level of amputation [8].

LEA is accompanied by a high risk of reulceration, reamputation, and death [9]. In patients with diabetes, perioperative mortality following LEA is high. Different studies found that early postoperative mortality (within 30 days) rates vary from 6% to 17% [10,11], and approximately 10% in-hospital mortality (IHM) in type 2 diabetes (T2DM) patients undergoing a major amputation was reported by our group [12]. Recently, Gurney et al. found that of the 2570 major LEAs in patients with diabetes that occurred between 2005 and 2014; 11.1% were followed by death within 30 days, and 17.6% were followed by death within 90 days [13].

Diabetes increases the risk of death in the longer term following amputation compared with patients without diabetes [1]. In the Netherlands, Fortington et al. [14] reported a 5-year mortality rate following the LEAs of 77% in patients with diabetes. Furthermore, Schofield et al. concluded that the presence of diabetes reduces survival by almost 50%, with a mean life expectancy of 27.2 months compared to 46.7 months in people with and without diabetes, respectively [15]. Furthermore, diabetes has also been shown to double the likelihood of requiring a second or contralateral amputation [16].

Another important factor associated with life expectancy is the level of amputation [17]. Lopez-Valverde et al. [18] reported that the survival rates at 1, 3, and 5 years were 90.6%, 72.8%, and 55.5% in patients with diabetes who underwent minor amputation, and 70.8%, 41.3%, and 34.4% in patients with diabetes who underwent major amputation, respectively.

Despite the relevance of this complication associated with diabetes, little information is available on long-term outcomes following diabetes-related LEAs in Spain [18].

The aims of our study were to assess the trend in the incidence of LEA among T2DM patients living in the Autonomous Community of Madrid from 2006 to 2015, and to compare the mortality risk in diabetic individuals who underwent LEA with that of age and sex-matched diabetic individuals who did not undergo LEA.

## 2. Materials and Methods

### 2.1. Design, Setting, and Participants

We conducted a descriptive observational study to assess the trend in the incidence of LEA and a retrospective cohort study to evaluate whether undergoing LEA is a risk factor for long-term mortality among T2DM patients.

In Spain, the National Health System (NHS) is a public health care insurance system with universal coverage and is fully financed by the general tax fund [19]. Regarding diabetic care, Spain offers a health coverage system with well-developed care, free at point of delivery. All diabetic expenses are fully covered by the NHS, with no out of pocket payments [20,21]. T2DM diabetes patients are mostly treated in a primary care setting, with varying participation from specialists, depending on available human resources, as well as local or regional policies [20,21]. In most regions, including Madrid, primary diabetes prevention plans are integrated into programs to prevent cardiovascular disease and obesity, and plans for healthy nutrition and habits. Early detection activities and diabetes education programs are also implemented [20,21]. However, the number of diabetic foot units in Spanish hospitals is still low and Rubio et al. reported that they only cover the foot care of one out of every four patients with diabetes [21]. Actually, the NHS does not recognize the right to diagnosis and treatment of foot pathologies by podiatrists and leaves the population the only alternative, for those who can afford it, to be treated in the private sector [20,21].

Data were obtained from the Hospital Discharge Database (HDD) for the Autonomous Community of Madrid (ACM). The HDD contains administrative and clinical data regarding all yearly admissions to public hospitals in the ACM. For each hospitalization, information available from the database and used in the present study included the hospital code; patient code, age, sex, admission and discharge dates; discharge status (dead, ordinary discharge, voluntary discharge, transferred to other hospital, or transferred to a social institution); main discharge diagnosis; up to 14 additional diagnoses; and up to 20 diagnostic and therapeutic procedures. The International Classification of Diseases—Clinical Modification, 9th edition (ICD-9-CM) is used for coding. The databases provided to us by the Health Authorities of the ACM were anonymous, but the individual patient code allowed us to link the records for the same patient over different years. Details on the HDD can be found elsewhere [22].

We analyzed hospital admissions from 1 January 2006 to 31 December 2015. The ACM is one of the 17 Autonomous Communities in Spain and had a population of 6,008,183 in 2006, which increased to 6,436,996 in 2015 [23].

In this study, only admissions of persons aged 40 years or over with ICD-9 codes for T2DM (250.x0 and 250.x2) in any of the 14 diagnostic positions were included. Those with a code for type 1 diabetes mellitus (250.x1 and 250.x3) were excluded.

### 2.2. Main Outcome Measures

The main study outcome measures were the incidence of LEA and mortality after LEA among T2DM patients compared with age and sex-matched T2DM individuals without LEA.

All hospital discharge records with an LEA code (ICD-9 codes: 84.10–84.19) in any of the 20 procedures’ fields were identified. We classified LEA, as proposed by Tseng et al. [24], into three levels: (i) minor, including toe (84.11), transmetatarsal (84.12–84.13), and distal transtibial (84.14) amputations; (ii) major below-knee (MBK) (transtibial; 84.15, 84.16) amputations; and (iii) major above knee (MAK) (transfemoral; 84.17–84.19) amputations.

To increase the probability of identifying the first LEA for each T2DM patient, those who had any of these codes in any diagnosis field of the HDD from 1 January 2003 to 31 December 2005 were excluded. All LEAs performed on a person with diabetes were considered diabetes-related.

If two or more amputations were coded during the same hospitalization, these were considered a single amputation because there are no variables in the HDD to enable us to assess whether the amputations were bilateral or contralateral and if the second or later amputations were a review of the previous. In this case, the highest anatomic level was assigned as a single procedure.

Patients were excluded if traumatic (895–897) or tumor-related peripheral amputations (170.7, 170.8) were codified.

The vital status of every patient up to 31 December 2015 and the date of death were obtained from the Spanish National Statistics Institute. Survival time was calculated from the date of the first amputation coded until the date of death or the end of the study period, whichever came first.

### 2.3. Cohort Study Design

We defined an exposed cohort for each year as all persons with T2DM who had a hospital discharge with LEA.

If the same patient had more than one discharge with LEA, we selected the first one as the level of amputation.

To create the unexposed cohort, for each subject in the exposed cohort, we selected a matched T2DM patient without LEA in the period 2003–2015 and an identical year of discharge, sex, age, and hospital of admission.

### 2.4. Study Variables

We analyzed age, sex, and the presence of the following clinical conditions in the hospital discharge data: myocardial infarction, congestive heart failure, cerebrovascular disease, dementia, chronic obstructive pulmonary disease (COPD), rheumatic disease, renal disease, any malignancy—including lymphomas, liver disease, PVD, and a metastatic solid tumor. We also calculated the number of conditions included in the Charlson comorbidity index (CCI) codified for each subject and categorized this variable in those with 1, 2, 3, or 4 or more conditions. The ICD-9 codes used to identify these clinical conditions and to calculate the CCI were those described by Quan et al. [25]. We also identified other pathogenesis that may have contributed to the explanation of survival differences, including neuropathy, Charcot foot, gangrene, infection, and dialysis. The ICD9 codes used to identify those conditions are those described by Hicks et al. [26]. Finally, we assessed the prevalence of obesity (ICD-9 codes: 278.00 and 278.01), hypertension (401, 401.0, 401.1, and 401.9), disorders of lipid metabolism (272.0-2 and 272.9) and tobacco use (305.1 and V15.82).

### 2.5. Statistical Analysis

For each year, the incidence rate was calculated as the ratio of the number of LEAs to Madrid´s resident population with T2DM. As a consequence, a patient could be included several times in the statistics if he/she had LEA two different times within one or subsequent years. The estimated population with T2DM per year was calculated using the Spanish National Health Surveys and the Di@bet.es study, as previously described by our group [27,28,29].

To test the incidence time trends, Poisson regression models were used separately for the three amputation levels, with sex and age being included in the model as independent variables.

Descriptive analysis was performed, with the distributions of variables compared among the exposed and unexposed cohorts and summarized using frequencies and means (SD). Comparisons were performed using the chi-squared statistic for proportions and Student’s *t*-tests for means.

We assessed survival with the Kaplan–Meier method, stratified for LEA status and according to the level of amputation. The log-rank test was used to compare the survival distributions.

After excluding patients who died within the first 30 days (perioperative mortality), multivariable Cox proportional hazards models for all-cause of death were performed, and hazard ratios (HRs) with 95% confidence intervals were estimated. A two-sided *p* < 0.05 was considered statistically significant. All statistical analyses were performed with Stata version 10.1 (Stata, College Station, Texas, USA).

## 3. Results

In the ACM from 2006 to 2015, the number of hospital discharges of patients aged 40 years or over with T2DM was 764,736. A code for LEA was found for 7945 (1.04%) hospitalizations, with 5245 (66.02%) being minor, 731 (9.20%) MBK and 1969 (24.78%) MAK.

### 3.1. Time Trends in the Incidence of LEA and Results of the Cohort Study

#### 3.1.1. Time Trends in the Incidence of LEA

Table 1 shows the trends from 2006 to 2015 in the numbers and incidence rates of LEA among T2DM patients living in Madrid (Spain). The number of minor LEAs increased from 408 in 2006 to 586 in 2015. The increase in the incidence was statistically significant (*p* = 0.007) after a Poisson regression analysis adjusted by sex and age was conducted.

The incidence rates of MBK decreased significantly from 24.9 to 17.1 per 100,000 T2DM individuals over time (*p* < 0.001). The same trend was observed for MAK, with the incidence decreasing from 63.9 to 48.2 per 100,000 T2DM individuals from 2006 to 2015 (*p* < 0.001).

The total number of LEAs rose from 693 in 2006 to 834 in 2015, but after Poisson regression analysis, the change over time was not significant (*p* = 0.063).

#### 3.1.2. Results of the Cohort Study

As shown in Table 2, the number of T2DM patients who underwent a first LEA was 5325 (exposed cohort), and we found a matched T2DM patient without LEA for each member of the exposed cohort.

The mean age of T2DM individuals with LEA was 70.16 years, and 72.54% were men. The most prevalent conditions among those with LEA were PVD (49%) and renal disease (20.79%). COPD (18.20%) and congestive heart failure (13.73%) were the two most frequent diseases among those without LEA.

Compared with the exposed cohort, those who were unexposed had higher prevalences of myocardial infarction, congestive heart failure, cerebrovascular disease, dementia, COPD, any malignancy—including lymphomas, liver disease, and metastatic solid tumors (all *p* < 0.05). On the other hand, those who underwent LEA had higher prevalences of rheumatic disease, renal disease, and PVD.

The proportion of T2DM patients with LEA who had a CCI of 3 or more was 34.93% compared with 25.58% among the unexposed cohort (*p* < 0.001).

Over the follow-up period, 48.58% of T2DM patients who underwent LEA died, compared with 34.05% of those without LEA (*p* < 0.001). However, the proportion that died within the first 30 days was higher among those without LEA (324/5325 (6.08%) versus 216/5325 (4.05%); *p* < 0.001).

**Figure 1:**
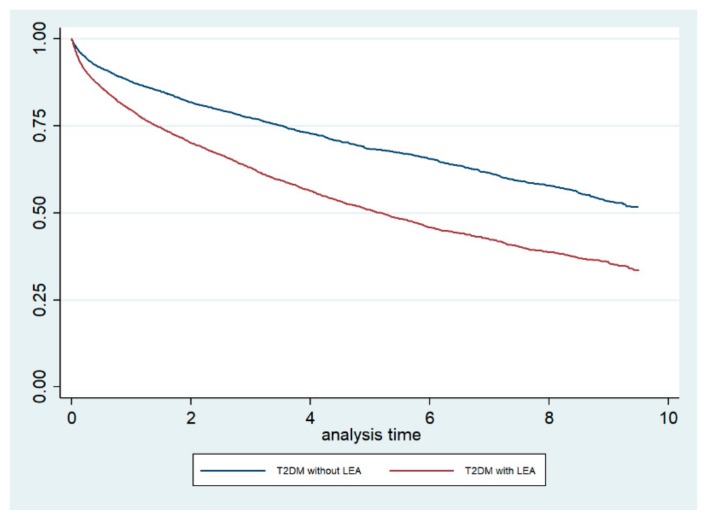
Kaplan–Meier survival curves, stratified for lower extremity amputation (LEA) status, among type 2 diabetic (T2DM) patients in the Autonomous Community of Madrid (Spain) from 2006 to 2015.

The Kaplan–Meier survival curves, stratified by LEA status, are shown in Figure 1. The log-rank test demonstrated that the crude mortality was significantly higher among T2DM patients who underwent LEA compared with those who did not undergo LEA (*p* < 0.001). Furthermore, when we conducted a Cox regression analysis including the CCI as a covariate, we obtained an adjusted HR for mortality of 1.75 (95% CI 1.65–1.87) for T2DM patients who underwent LEA.

**Figure 2:**
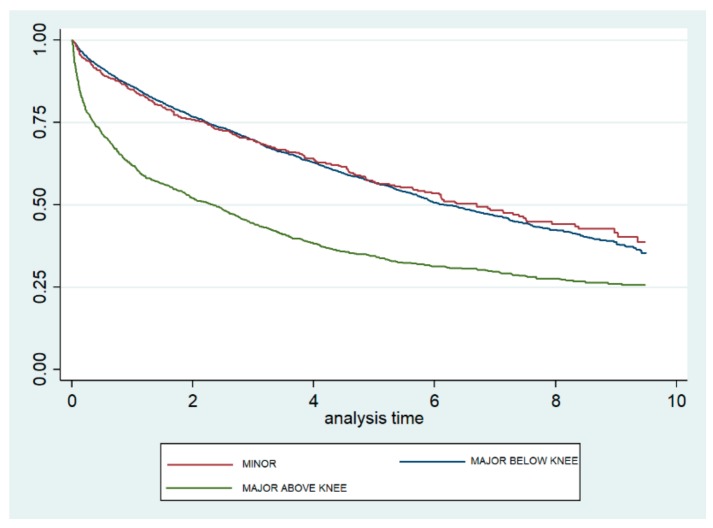
Kaplan–Meier survival curves, according to level of lower extremity amputation, among type 2 diabetic patients in the Autonomous Community of Madrid (Spain) from 2006 to 2015.

Figure 2 shows the Kaplan–Meier survival curves according to the level of amputation. The curves for minor and MBK amputations are very similar and show a significantly lower mortality than does the curve observed for MAK. The log-rank test yielded no significant difference between minor and MBK (*p* = 0.795) amputations, but did show a significant difference between minor and MAK (*p* < 0.001) amputations.

When only patients who underwent LEA were analyzed with a Cox regression model, we found that males had a significantly higher risk of dying than females (HR 1.13; 95% CI 1.04–1.24), and the risk increased as the CCI increased (HR 2.16; 95% CI 1.51–2.46 for those with 4+ versus one condition). Finally, using minor LEA as the reference category, the mortality was not significantly different for those with MBK amputations but was 1.34 times higher for those who underwent MAK amputations (95% CI 1.22–1.46) (Table 3).

Shown in Supplementary Table S1 are the trends in the Charlson comorbidity index, specific clinical conditions, and lifestyles among T2DM patients who underwent a LEA in the Autonomous Community of Madrid from 2006 to 2015. We observed a significant increment in the prevalence of PVD, neuropathy, gangrene, and infection, with no change over time for Charcot foot, chronic renal disease without dialysis, and dialysis. Regarding lifestyles, no significant variations over time were found for hypertension, disorders of lipid metabolism, or tobacco use; however, the prevalence of obesity increased significantly.

The results of the Cox regression models, including the clinical conditions that were identified in the pathogenesis of LEA in T2DM patients and the amputation level, are shown in Supplementary Table S2. Besides the level of amputation, suffering chronic renal disease without dialysis or having dialysis significantly increased the risk of dying in the follow up period. PVD was identified as a bad prognosis factor only for those who underwent a minor amputation.

## 4. Discussion

In our investigation, we found different trends over the last 10 years in the incidence rates of LEA among T2DM patients. There has been an increase in the number of T2DM patients undergoing minor LEA; however, a significant decrease was observed in MBK and MAK LEAs. For all LEAs, the trend was stable over the study period. These trends agree with the findings of previous studies conducted in Spain [3,12], and they are consistent with the findings of studies performed in Europe and other high-income countries [30,31,32,33,34]. 

Several studies have been published analyzing the incidence of amputations in different countries [12,30,31,32,33,34,35,36]. However, the results are difficult to compare because these studies use different coding mechanisms and data sources [12,30,31,32,33,34,35,36]. In year 2016, Carinci et al. published LEA rates in diabetes from 2000 to 2011 (age-sex standardized rates per 100,000 population aged 15 or over) in countries belonging to the Organization for Economic Cooperation and Development (OECD), providing data from 26 countries showing significant differences in the rates [35]. According to this investigation, for the year 2009, the lowest rate was found in Hungary (0.7 per 100,000 population aged 15 or over) and the highest in Germany (20.6). The equivalent figure for Spain that year was 10.6, with 14 countries showing lower values, and seven countries, higher values. Outside Europe is extreme, given the high rates in the USA (17.1 in 2010) and Israel (19.5 in 2010), and the low rates in New Zealand (6.7 in 2011). Overall, standardized LEA experienced a 12-year decline of over 40%, from a mean of 13.2 amputations per 100,000 in 2000 to a mean of 7.8 amputations per 100,000 in 2011 [35]. The influence of the different funding mechanisms of the health care system on the incidence of LEA among diabetic patients was also analyzed, and the results suggested lower amputation rates in health systems financed by public taxation when compared with insurance-based systems. The average difference in favor of tax-based versus insurance-based systems is equal to 4.5 per 100,000 diabetic amputations. In any case, as commented before, the authors remark that differences in methodological approaches across studies can lead to limited comparability [35].

Besides the characteristics of the health systems, studies conducted in several countries have found that different systems of diabetic care affect the incidence of LEA [21,37,38,39,40,41]. In Germany, the number of major amputations decreased by 19.0% from 2007 to 2011, and the ratio of major amputation/inpatients with diabetes (x 1000) also decreased from 8.44 to 5.75. Within that time period, the number of podiatrist foot care treatments per 1000 insured rose from 22 in 2007 to 60 in 2011 [37]. In the US, regions with the most intensive vascular care have the lowest amputation rate [39]. Another study showed that foot care in patients suffering from diabetes mellitus has improved, as podiatrist foot care can be prescribed by doctors and is funded by social health insurance [38]. In the South West region of England, Paisey et al. found that major diabetes-related lower limb amputation incidence is significantly inversely correlated with foot care services’ provision, concluding that the introduction of more effective service provisions resulted in significant reductions in major amputation incidence within 2 years [40]. In the UK, a sustained reduction in diabetes-related lower limb amputation was observed after the institution of job-planned, multidisciplinary, secondary care diabetic foot clinics [41].

In Spain, the introduction of a multidisciplinary team, coordinated by an endocrinologist and a podiatrist, for managing diabetic foot disease, was associated with a reduction in the incidence of major amputations in patients with diabetes [21]. 

These reports agree that the successful introduction of service improvements reduces the incidence rates of diabetic foot ulcers and prevents avoidable amputations, thus recommending the integration of all diabetes foot care services.

A remarkable result of our study is the relative increase in the number of minor LEAs and the decrease in major LEAs. Different studies have reported that the suspected drivers behind this shift from major to minor amputations are improvements in preventive health care structures associated with the performance of more conservative limb-salvaging procedures and increasingly more aggressive treatment of PVD in these patients [42,43]. Furthermore, new surgical techniques and nonsurgical therapies may be contributing to these changes [35]. Another possible explanation is that this shift may be partly a consequence of the improved glycemic control, comorbidity treatments, and changes in blood pressure or lipid profiles. Regarding glycemic control, the HDD does not include laboratory results, so it is not possible to assess this issue. However, studies conducted in our country using the Primary Care Electronic Population Database have shown there were no relevant changes in glycemic control from 2007 to 2013, finding no obvious trend towards an increase in the proportion of patients with an adequate HbA1c target whatever the cut-off used, and the mean HbA1c values did not significantly change over time regardless of the treatment step [44]. As can be seen in Supplementary Table S1, the prevalence of hypertension, lipid disorders, and tobacco use has not changed over time among T2DM patients undergoing LEAs, so the possible effect of the improvement of these lifestyles on the level of LEAs cannot be confirmed in our population.

In our study, the presence of comorbidities in T2DM patients who underwent LEA, including PVD and renal disease, was more common than in those who were unexposed. A recent meta-analysis of risk factors for amputation in patients with diabetic foot infections reported that PVD was associated with a 2.35-fold increase in the risk of amputation (95% CI, 1.484–3.718). Other comorbidities, such as hypertension, cerebrovascular disease, coronary artery disease, and chronic renal failure, showed no statistically significant predictive ability for amputations in patients with diabetic foot infections [45]. However, different stages of nephropathy, including microalbuminuria and macroalbuminuria, have also been reported to play a role in the development of DFUs [46], thus contributing indirectly to the LEA risk.

We found that undergoing LEA increases the risk of death by 75%. However, the early mortality (within 30 days) was higher in non-LEA patients, which indicates that patients who underwent LEA might have had better surgical and perioperative health care and a better control over their diabetes via strict glycemic control [30,47], although if they survived the first month, over time, patients who underwent LEA died more often than those who did not. In the UK, a cohort study using data from The Health Improvement Network evaluated those with LEA to those without over time and concluded that individuals with diabetes and LEA were more likely to die at any given point in time than those who had diabetes but no LEA. One explanation is that the risk of death is related to those being selected for LEA, which is a procedure commonly viewed as a last effort to treat a patient’s foot ulcer. However, the authors indicated that it was likely that the major causes of death in those with diabetes and LEA were similar to the causes in those who had not undergone LEA (e.g., myocardial infarction, cerebrovascular disease, congestive heart failure, and chronic kidney disease) [48].

The amputation level is also an important factor in predicting the mortality of amputees. Most of the previous studies agreed that major amputees have higher mortality than do minor amputees [18,49]. In our study, when comparing the level of amputation, we observed that the risk of death was significantly higher in patients undergoing MAK than in those undergoing the other LEA procedures (minor and MBK). A systematic review showed that the 5-year mortality after LEA ranged from 29% to 69% following minor amputations [9]. However, after a major amputation, mortality worsened. Data from patients with diabetes who underwent major amputations were associated with worse outcomes: 77% [9] and 85.7% in cases of MAK amputations [50].

Different studies found that the rate of amputation was higher in men than in women [51]; however, female sex is a predictor of in-hospital mortality after LEA [3]. In our cohort, we found that male sex was associated with higher mortality after LEA in patients with diabetes. Gurney et al. [13] found little difference between the sexes in terms of the adjusted risk of postoperative mortality (90 days) and concluded that female patients who undergo amputation are similar to their male counterparts in terms of the underlying risk factors for postoperative mortality. However, it is possible that male patients have risk factors, such as tobacco use, high cholesterol or worse diabetes control, which are variables that are not collected in the HDD used in our investigation, and this could explain their higher mortality.

Our data indicate that older age and comorbidities were independently associated with higher mortality after LEA in patients with diabetes. These findings are similar to those reported in other studies that observed differences in postamputation mortality outcomes between younger and older patients [48], indicating that the risk of postoperative mortality is the greatest among the elderly population [13].

Comorbid conditions, especially macrovascular disease, are more common among patients undergoing amputation [16]. The leading cause of mortality in patients with diabetes is cardiovascular events. Patients who needed major amputations suffered from more severe cardiovascular disease than others (median survival 40 months in subjects with cardiovascular events versus 61 months in those without cardiovascular events). Therefore, the attributed mortality of those undergoing major amputations is higher because of a more complex generalized disease [52].

Previous studies identified diabetic nephropathy as a risk factor for significantly lower long-term survival after LEA. Hoffmann et al. [52] found that the median survival in patients with diabetic nephropathy was 27 months, while the median survival in those without diabetic nephropathy was 79 months. Assi et al. found that the presence of chronic kidney disease prior to amputation was associated with worse long-term survival (OR 2.27; 95% CI 2.02–5.06) [53]. Those results agree with ours, showing that chronic renal disease, with or without dialysis, increases the risk of dying aside from the level of amputation. Beyaz et al. reported that hemo-dialysis increases mortality by 1.53 times (95% CI, 1.218–1.936) among diabetic patients undergoing below-knee amputations [54].

In our investigation obesity was not associated with mortality after LEA, even if the hazard ratios were bellow one (suggesting a protective effect) in all levels of amputation. Some authors have suggested that the “obesity paradox” may exist for LEAs in the diabetic population [55].

Future research should include understanding the factors driving changes in trends in the incidence of LEA. These factors include, at the individual-level, clinical conditions and health policies, and organizational characteristics. Identifying those variables would help to prioritize preventive approaches [20,36]. It is also necessary to identify new prognostic factors for mortality after LEA. Recently Ikura et al. found that brachial–ankle pulse wave velocity, but not ankle-brachial index, could be a predictor for all-cause mortality in diabetic patients after LEA [56]. 

In Spain, investigation is needed to ascertain the position of diabetes in the Spanish national health care system. This will require improved data collection to monitor incidence, prevalence, expenditure, and outcomes. Studies are also needed to understand the disparities in access to care; in particular, the access to revascularization procedures, diabetic foot units, podiatrist care, diabetes care planning, and educational provisions [20]. Finally, any future research needs to employ internationally agreed protocols, and provide better documentation of the type of diabetes, disease duration, pharmacological therapy, and systemic complications [33].

As with all epidemiological studies, our study has several potential limitations. We used the HDD, which is an administrative database rather than a clinical database [22]. Therefore, it was subject to coding errors. In addition, it is based on mandatory discharge data in the ACM, and it lacks important clinical details, such as the duration of diabetes, diabetes complications, and treatments or laboratory results, which are factors that have been associated with mortality after LEA. Furthermore, relevant variables, such as BMI, are not included in the HDD. However, Buckley et al. detected high levels of agreement between hospital discharge data and medical records for LEA and diabetes, and suggested that hospital discharge data are sufficiently reliable to monitor trends in LEAs in people with diabetes [57]. Finally, our study only includes LEAs conducted in public hospitals, so we lack information on the small number of surgeries undergone in private clinics. 

## 5. Conclusions

Our data show a decrease in the incidences of major below-knee and major above-knee amputations in patients with T2DM; however, an increase was found in those undergoing minor LEAs. Undergoing LEA increases the risk of death by 75%. Older age, male sex, and comorbidities are responsible for mortality in in patients with T2DM who have undergone LEA. Long-term survival was worse in patients who underwent major above-knee LEAs than in those who underwent other types of LEA. These results may suggest an improvement in the quality of diabetic foot care in recent years, but additional improvements in preventive care and early treatment for patients with diabetes, and the aggressive treatment of comorbidities, are needed to reduce LEA further.

## Figures and Tables

**Table 1:** Trends 2006–2015 in lower extremity amputations (LEAs) among type 2 diabetes mellitus (T2DM) patients in the Autonomous Community of Madrid.

Level of Amputation		2006	2007	2008	2009	2010	2011	2012	2013	2014	2015	*p*
Minor	N	408	436	488	506	540	563	564	564	590	586	0.007
	Incidence	127.3	136.0	146.7	147.0	156.8	151.8	141.9	144.0	152.9	154.2	
Major. Below Knee	N	80	85	84	80	72	65	91	54	55	65	<0.001
	Incidence	24.9	26.5	25.2	23.2	20.9	17.5	22.9	13.8	14.2	17.1	
Major. Above knee	N	205	208	208	212	184	195	191	206	177	183	<0.001
	Incidence	63.9	64.9	62.6	61.5	53.4	52.6	48.0	52.6	45.9	48.2	
Total	N	693	729	780	798	796	823	846	824	822	834	0.062
	Incidence	216.1	227.3	234.6	231.7	231.2	221.9	212.8	210.4	213.0	219.5	

Incidence per 100,000 T2DM individuals living in Madrid that year. *p* for time trend used Poisson’s regression models adjusted by sex and age.

**Table 2:** Characteristics of T2DM patients who underwent a LEA and matched T2DM patients hospitalized without LEA.

		With Amputation (5325)		Without Amputation (5325)		
		*N*	%	*n*	%	*p*
Gender	Male	3863	72.54	3863	72.54	NA
	Female	1462	27.46	1462	27.46	
Age. Mean (SD)		70.16	(12.84)	70.16	(12.84)	NA
Myocardial infarction		345	6.48	409	7.68	0.016
Congestive heart failure		563	10.57	731	13.73	<0.001
Cerebrovascular disease		484	9.09	589	11.06	0.001
Dementia		179	3.36	203	3.81	0.001
Chronic obstructive pulmonary disease		570	10.70	969	18.20	<0.001
Rheumatic disease		111	2.08	72	1.35	0.003
Chronic Renal disease		1107	20.79	581	10.91	<0.001
Any malignancy, including lymphomas		161	3.02	514	9.65	<0.001
Liver disease		236	4.43	476	8.94	<0.001
Metastatic solid tumour		31	0.58	199	3.74	<0.001
Peripheral vascular disease		2609	49.00	336	6.31	<0.001
Charlson comorbidity index	1	1386	26.03	1904	35.76	<0.001
	2	2079	39.04	2059	38.67	
	3	1290	24.23	1002	18.82	
	4+	570	10.70	360	6.76	
Died in the follow up period		2587	48.58	1813	34.05	<0.001
Mortality in time periods	<30 days	216	8.35	324	17.87	< 0.001
	30 days - 23 months	1293	49.98	751	41.42	
	24 months - 47 months	556	21.49	339	18.70	
	48 months - 83 months	397	15.35	281	15.50	
	≥ 84 months	125	4.83	118	6.51	

**Table 3:** Factors associated with dying among T2DM patients who had undergone a LEA.

	Cox	Hazard Ratio *	Lower Limit	Upper Limit
age in years		1.037	1.03	1.04
gender	female	1		
	male	1.13	1.04	1.24
Charlson comorbidity index	1	1	1	1
	2	1.23	1.10	1.38
	3	1.71	1.53	1.93
	4+	2.16	1.51	2.46
amputation level	minor			
	major below knee	1.06	0.91	1.22
	major above knee	1.34	1.22	1.46

* Hazard ratios obtained using multivariable Cox regression model.

## Supplementary Materials

The following are available online at https://www.mdpi.com/2077-0383/8/10/1597/s1, Table S1: Trends 2006-2015 in Charlson comorbidity index, specific clinical conditions and lifestyles among T2DM patients who have undergone a lower extremity amputation in the Autonomous Community of Madrid, Table S2: Factors associated with dying among T2DM patients who have undergone a lower extremity amputation according to amputation level.
