# Supplementary material for: Time Trends in the Incidence of Long-Term Mortality in T2DM Patients Who Have Undergone a Lower Extremity Amputation. Results of a Descriptive and Retrospective Cohort Study
Source: J Clin Med. 2019 Oct 2;8(10):1597. doi: 10.3390/jcm8101597 (PMC6832955; doi:10.3390/jcm8101597)
Supplement: Supplementary file 1 [file jcm-08-01597-s001.pdf]

Supplementary Table S1. Trends 2006-2015 in Charlson comorbidity index, specific clinical conditions and lifestyles among T2DM patients who have undergone a lower extremity amputation in the Autonomous Community of Madrid.

	**2006**	**2007**	**2008**	**2009**	**2010**	**2011**	**2012**	**2013**	**2014**	**2015**	**Total**	**p-value**
CCI,* Mean (SD)	1.52(0.73)	1.52(0.75)	1.51(0.67)	1.53(0.72)	1.48(0.72)	1.53(0.73)	1.53(0.74)	1.5(0.72)	1.53(0.7)	1.54(0.74)	1.52(0.72)	0.556
Peripheral vascular disease, N(%)	295(53.15)	274(48.24)	233(43.96)	222(44.05)	291(49.07)	262(49.53)	260(49.06)	274(51.12)	241(46.98)	257(55.03)	2609(49)	0.006
Neuropathy, N(%)	75(13.51)	73(12.85)	52(9.81)	59(11.71)	105(17.71)	91(17.2)	107(20.19)	88(16.42)	80(15.59)	74(15.85)	804(15.1)	<0.001
Charcot foot, N(%)	1(0.18)	4(0.7)	4(0.75)	4(0.79)	6(1.01)	5(0.95)	7(1.32)	3(0.56)	3(0.58)	2(0.43)	39(0.73)	0.656
Gangrene(%)	263(47.39)	267(47.01)	241(45.47)	230(45.63)	307(51.77)	279(52.74)	240(45.28)	257(47.95)	279(54.39)	241(51.61)	2604(48.9)	0.010
Infection, N(%)	97(17.48)	106(18.66)	136(25.66)	117(23.21)	178(30.02)	161(30.43)	162(30.57)	149(27.8)	161(31.38)	144(30.84)	1411(26.5)	<0.001
Chronic renal disease without dialysis ,N(%)	76(13.69)	70(12.32)	74(13.96)	69(13.69)	71(11.97)	84(15.88)	70(13.21)	89(16.6)	67(13.06)	81(17.34)	751(14.1)	0.169
Dialysis, N(%)	45(8.11)	37(6.51)	30(5.66)	30(5.95)	37(6.24)	43(8.13)	41(7.74)	48(8.96)	49(9.55)	39(8.35)	399(7.49)	0.195
Obesity, N(%)	44(7.93)	45(7.92)	50(9.43)	39(7.74)	69(11.64)	61(11.53)	53(10)	66(12.31)	78(15.2)	75(16.06)	580(10.89)	<0.001
Hypertension, N(%)	376(67.75)	367(64.61)	340(64.15)	319(63.29)	397(66.95)	361(68.24)	354(66.79)	352(65.67)	351(68.42)	294(62.96)	3511(65.93)	0.480
Disorders of lipid metabolism, N(%)	252(45.41)	256(45.05)	232(43.71)	212(41.88)	264(44.52)	229(43.29)	224(42.26)	249(46.46)	235(45.92)	202(43.89)	2358(44.28)	0.369
Tobacco use ,N(%)	223(40.28)	236(41.75)	225(42.36)	202(40.11)	242(40.81)	223(42.16)	232(43.77)	220(47.03)	220(42.88)	203(43.25)	2226(41.80)	0.168

CCI: Charlson comorbidity index excluding chronic Peripheral vascular disease, Chronic Renal disease and dialysis. P for time trend using Poisson regression models adjusted by sex and age.

Supplementary table S2. Factors associated with dying among T2DM patients who have undergone a lower extremity amputation according to amputation level.

	**Cox**	Minor amputation	Major Below Knee amputation	Major Above Knee amputation	**All amputation**
		Hazards ratio (95%cCI)	Hazards ratio (95%cCI)	Hazards ratio (95%cCI)	Hazards ratio (95%cCI)
Age in years		1.03 (1.03-1.04)	1.03(1.01-1.05)	1.02 (1.01-1.03)	1.03(1.03-1.04)
Gender	Female	1	1	1	1
	Male	1.08(0.93-1.26)	1.46(0.85-2.50)	1.32(1.05-1.65)	1.21(1.07-1.36)
Charlson comorbidity index*	1	1	1	1	1
	2	1.35(1.17-1.56)	1.22(0.84-1.77)	1.43(1.14-1.79)	1.38(1.23-1.55)
	3	1.56(1.25-1.95	1.47(0.58-3.67)	1.42(1.05-1.91)	1.51(1.27-1.78)
	4+	2.70(1.58-4.60)	0.43(0.56-3.25)	1.61(0.59-4.38)	1.91(1.21-3.02)
Peripheral vascular disease	No	1	1	1	1
	Yes	1.28(1.11-1.46)	0.99(0.68-1.45)	1.12(0.89-1.41)	1.22(1.09-1.36)
Neuropathy	No	1	1	1	1
	Yes	1.11(0.94-1.32)	1.42(0.88-2.30)	0.94(0.64-1.38)	1.09(0.94-1.26)
Charcot foot	No	1	1	1	1
	Yes	0.91(0.43-1.94)	0.74(0.18-3.15)	7.05(0.93-53.60)	0.98(0.52-1.84)
Gangrene	No	1	1	1	1
	Yes	1.09(0.96-1.25)	1.10(0.76-1.60)	1.03(0.83-1.26)	1.09(0.98-1.21)
Infection	No	1	1	1	1
	Yes	1.05(0.91-1.20)	0.98(0.66-1.43)	1.10(0.81-1.49)	1.03(0.91-1.16)
Chronic renal disease without dialysis	No	1	1	1	1
	Yes	1.39(1.17-1.67)	1.72(1.09-2.72)	1.39(1.05-1.83)	1.41(1.23-1.63)
Dialysis	No	1	1	1	1
	Yes	1.90(1.51-2.40)	2.85(1.66-4.90)	1.37(0.93-2.04)	1.85(1.54-2.23)
Obesity	No	1	1	1	1
	Yes	0.80(0.31-2.13)	0.92(0.75-2.26)	0.89(0.62-1.43)	0.88(0.67-1.36)

Amputation level	Minor	NA	NA	NA	1
	Major Below Knee	NA	NA	NA	1.04(0.87-1.24)
	Major Above Knee	NA	NA	NA	1.30(1.15-1.47)

Charlson comorbidity index excluding chronic Peripheral vascular disease, Chronic Renal disease and dialysis. Hazard ratios obtained using multivariable Cox regression models. NA Not applicable
